# Youth visiting the emergency department after a suicide attempt, suicidal ideation or non-suicidal self-injury: trends, repeat visits and costs

**DOI:** 10.1192/bjo.2026.11031

**Published:** 2026-04-28

**Authors:** Naomi van der Linden, Leona Hakkaart-van Roijen, Kinke Lommerse, Merel van Loon-van Gaalen, Shanna van der Linden, Yvonne Bal, Christien van der Linden

**Affiliations:** Institute for Health Systems Science, Delft University of Technology, Delft, The Netherlands; Erasmus School of Health Policy & Management, Erasmus University Rotterdam, Rotterdam, The Netherlands; Psychiatry Department, Haaglanden Medical Centre, The Hague, The Netherlands; Research Department, 113 Suicide Prevention, Amsterdam, The Netherlands; Emergency Department, Haaglanden Medical Centre, The Hague, The Netherlands; Parnassia Group, Sumona Indigo Haaglanden, The Hague, The Netherlands; Acute Care Division, Haaglanden Medical Centre, The Hague, The Netherlands

**Keywords:** Suicide, self-harm, youth mental health, emergency care, costs

## Abstract

**Background:**

In The Netherlands, it is unknown whether the number of youth suicide-related emergency department visits has changed over time. Also, insight is needed in the hospital costs for managing these patients, as a first step toward the economic evaluation of suicide prevention measures.

**Aims:**

This study examines (a) changes in emergency department-recorded suicide attempts, suicidal ideation and non-suicidal self-injury in youth, including repeat emergency department visits; and (b) related hospital costs for these patients, from a health insurer perspective.

**Method:**

In this cross-sectional study, data from various sources was combined to identify all youth aged ≤27 years visiting a Dutch inner-city emergency department between 2016 and 2023 for a suicide attempt, suicidal ideation or non-suicidal self-injury. Hospital records were reviewed manually to determine inclusion. Ambiguities were discussed within an expert panel and descriptive analyses, Poisson regression and logistic regression analyses were performed. For a subset of 30 patients, invoiced costs were determined.

**Results:**

The number of suicide attempts increased by approximately 5% annually, peaking in 2022 (*n* = 172); there were significantly more female patients (71%), and the median age was 21 years. Cases of suicidal ideation showed a similar trend, whereas the number of recorded non-suicidal self-injuries reduced. A total of 28.5% of all patients (*n* = 281) had one or multiple repeat visits for the above reasons. Median suicide attempt-related costs per case were €930, range €385–€33 473.

**Conclusions:**

Since 2016, an increasing number of youth visited the emergency department of a Dutch hospital after a suicide attempt, but this increase does not seem to continue after 2022. Hospital-invoiced costs differ substantially between patients.

Worldwide, suicide is the fourth leading cause of death among 15**–**29 year olds.^1^ In The Netherlands, suicide is the leading cause of death for young people (<30 years),^2^ and the number of suicide deaths among this group is increasing. Between 2013 and 2023, the number of suicides in this group increased from 243 in 2013 to 300 in 2023.^3^ In 2022, 67 children (<20 years) and 241 young adults (20–29 years) in The Netherlands died by suicide.^4^ The numbers of suicide attempts and non-suicidal self-injuries are substantially higher, but exact numbers are lacking because there is no registration on a national level. Schweren et al discuss the challenges in registering non-fatal suicide attempts on a national level.^5^ However, we know that increasing numbers of young people in The Netherlands have suicidal thoughts: in early 2023, 14.1% of people aged 12**–**25 years had suicidal thoughts, compared with 8.5% at the end of 2021.^6^ Prior research in England has shown that a substantial proportion of suicide attempts (13.6%) lead to a repeat event within 1 year.^7^ Costs of care for suicidal youths are currently unknown in The Netherlands.

Key groups of identified risk factors in youth suicide traditionally include individual factors (e.g. mental health or behavioural difficulties, prior suicidal behaviour), family-related factors (e.g. family psychopathology, parental divorce) and socio-environmental and contextual factors (e.g. school-related factors such as bullying).^8,9^ A discussion on risk factors for suicide in youth is beyond the scope of this paper, but Hughes et al^10^ list a range of useful review papers on this topic. In some cases, poor accessibility of appropriate youth mental healthcare in The Netherlands may worsen the condition of youths with suicidal thoughts.^9^ In 2021, the Health and Youth Care Inspectorate reported that youth mental healthcare was insufficient in many regions of The Netherlands. Certain regions reported an increasing number of mental health crisis situations and long waiting times before receiving mental healthcare, even after repeated suicide attempts.^11^ One in five youths under 20 years who died by suicide in 2017 in The Netherlands were on the waiting list to receive care.^9^ Despite regional collaborations and a focus on reducing waiting lists, the Inspectorate and the Dutch Healthcare Authority reported in 2023, that no improvements had been observed.^12^

International literature recognises the emergency department as a valuable setting for initiating suicide prevention measures, such as active contact and follow-up to reduce the risk of a repeat suicide attempt for patients admitted to emergency departments after a suicide attempt.^13,14^ To inform emergency department initiatives and other youth suicide prevention efforts, it is important to understand both the scope of the problem and the way it develops over time. Given the lack of national and regional data on suicide attempts, local emergency department data is one of the few sources to provide insights into trends in the number and type of youth suicide attempts. However, there are challenges in reliably recording and retrieving these data. These challenges include the lack of a single code or description for recording suicide attempts, complicating automatic data retrieval. Additionally, the definition of a suicide attempt requires the patient to have had an intention to die, but in the emergency department setting, this is often unclear.^15^

This study is designed to overcome these challenges by combining data from different sources, to determine:

- changes in the number and patient characteristics (age, gender) of youth suicide attempts, suicidal ideation and non-suicidal self-injuries presenting at the emergency department over time;
- changes in the number of repeated suicide attempts by youths presenting at the emergency department, compared with repeat visits by patients of the same age group for other reasons;
- insight into the costs of the hospital care provided to youths visiting the emergency department after a suicide attempt;
- to what extent the combination of various data sources are suitable for identifying youth suicide attempts presenting at the emergency department.

The clinical findings of this study can be used to optimise future emergency department initiatives and other interventions for suicide prevention in youths. The economic findings, although limited in scope, aim to direct future research and economic evaluations in this space.

## Method

### Setting

The Haaglanden Medical Center (HMC) is a top-rated, clinical, inner-city hospital situated in The Hague, The Netherlands. The Hague is a diverse city, both socioeconomically and ethnically, with a high number of migrants and lower-income groups and a significant representation of Moroccan, Turkish and Surinamese communities.^16,17^ The HMC emergency department functions as a regional level 1 trauma and acute neurovascular centre, receiving approximately 50 000 patient visits annually, with a 30% admission rate.

### Population

Patients were eligible for inclusion if they fulfilled the criteria specified in Table 1. Supplementary Table 1 provides additional information (diagnosis codes) on these criteria. Resulting demographic and clinical characteristics of these patients can be found in the Results section and Supplementary Table 4.

**Table 1:** Eligibility criteria

Study population	Inclusion criteria	Clarification
For all included patients	Age ≤27 years	Aligning with a prior suicide prevention project for youth at the emergency department
	Visited the emergency department in the study hospital from 1 January 2016 until 31 December 2023	Suicide-related visits to other hospitals within the study period were excluded, even if they were mentioned in the records of the study hospital
For cases recorded as suicide attempts	Visited the emergency department for physical harm inflicted by themselves, with at least some recorded intention to die	If the patient no longer expressed a willingness to die at the emergency department, but they had expressed it before (e.g. to the police) in the period from (just) before the incident up to and including the emergency department visit, the case was also registered as a suicide attempt
For cases recorded as suicidal ideation	Visited the emergency department without self-inflicted physical harm, but did express a wish to die	
For cases recorded as non-suicidal self-injuries	Visited the emergency department for physical harm inflicted by themselves, without the intention to die	This excludes alcohol intoxications without the intention of self-harm

Patients were excluded if they had objected against the use of their medical data for scientific research (hospital-level opt-out); or if hospital records were unavailable, e.g. in cases where patients were referred directly from triage in the emergency department to a general practitioner cooperative (resulting in no medical records).

**Table 2:** Data sources

Data source	Characteristics
Management data	List of emergency department patients registered manually by the emergency department manager since 2015. Inclusion criteria differed over time, including suicide attempts, non-suicidal self-injuries and intoxications (irrespective of intention). These management data were collected at the request of the hospital psychiatrist to assess the volume and characteristics of patients with psychiatric presentations. The original aim was to evaluate the need for hospital-based psychiatric care, including care in the medical psychiatric unit, which was installed in 2012.
Electronic health record data (CTcue)	The ‘Patient Finder’ tool from CTcue (version 4.13 for Windows; IQVIA, Durham, North Carolina, USA; =https://demo.pfs.solutions.iqvia.com/login?redirectTo=/projects) was used to identify eligible patients from the hospital electronic health records. All patients with an emergency department visit during the study period and a relevant ICD code were selected. The list of relevant codes was made based on reviewing prior literature^18–21^ and included diagnoses related to (attempted) suicide, suicidal ideation, suicidal tendencies and non-suicidal self-injury, but also codes for, among others, poisoning, asphyxia, psychiatric illnesses and specific types of injuries (e.g. ‘open wound of the forearm’). A full list of diagnosis codes is provided in the Supplementary Material.
Administrative data from the business intelligence department	Overviews of all emergency department visits from 2016 to 2023 were used to identify repeat visits and to detect additional inclusions that were missed in the other sources. These data integrate clinical and financial information. Cases were identified through a targeted screening of the triage flow chart ‘Overdose/intoxication’, since this was the most commonly used triage flow chart in attempted suicides.

**Table 3:** Poisson regression results for suicide attempts, using the natural logarithm of the total number of emergency department visits in the same age group as an offset

Parameter	*B*	s.e.	95% Wald confidence interval (lower; upper)	Hypothesis test Wald *χ* ^2^	d.f.	Significance	Exp(*B*)	95% Wald confidence interval for exp(*B*) (lower to upper)
Evaluation of yearly trend, using a continuous year variable								
Intercept	−98.822	27.686	−153.086 to −44.558	12.740	1	<0.001	<0.001	3.277E-67–4.454E-20
Year	0.047	0.014	0.020–0.074	11.728	1	<0.001	1.048	1.020–1.077
Evaluation of year-on-year incidence rate rations, comparing individual years against a reference (2016)								
Intercept	−4.217	0.099	−4.412 to −4.022	1795.989	1	<0.001	0.015	0.012–0.018
2023	0.381	0.127	0.132–0.631	8.966	1	0.003	1.464	1.141–1.880
2022	0.393	0.125	0.148–0.639	9.847	1	0.002	1.482	1.159–1.895
2021	0.281	0.132	0.022–0.541	4.506	1	0.034	1.325	1.022–1.717
2020	0.061	0.140	−0.215 to 0.337	0.188	1	0.665	1.063	0.807–1.400
2019	−0.112	0.139	−0.385 to 0.161	0.648	1	0.421	0.894	0.680–1.175
2018	0.270	0.133	0.009–0.531	4.113	1	0.043	1.310	1.009–1.700
2017	0.226	0.134	−0.037 to 0.489	2.847	1	0.092	1.254	0.964–1.630
2016	0	–	–	–	–	–	1	–

**Table 4:** Trends in number of youth suicide attempts, suicidal ideation and non-suicidal self-injuries

Year	Suicide attempts			Suicidal ideation			Non-suicidal self-injuries			Rest emergency department visits by youth (12–27 years)		
	Number	% female	Median age, years (range)	Number	% female	Median age, years (range)	Number	% female	Median age, years (range)	Number	% female	Median age, years (range)
2016	101	72.5	21 (14–27)	6	50.0	20 (13–27)	34	66.7	22 (14–27)	6708	47.4	21 (12–27)
2017	124	78.9	23 (13–27)	12	33.3	23 (19–27)	29	83.3	18 (14–27)	6541	46.7	21 (12–27)
2018	128	55.0	22 (14–27)	29	23.1	20 (12–27)	25	77.8	20 (16–27)	6446	46.8	22 (12–27)
2019	105	75.6	21 (13–27)	20	16.7	21 (17–23)	30	77.8	21 (15–26)	7813	46.4	21 (12–27)
2020	101	74.4	20 (14–27)	14	80.0	22 (16–27)	18	50.0	19 (15–25)	6310	44.8	21 (12–27)
2021	131	72.2	21 (13–27)	32	72.7	21 (15–27)	23	55.6	22 (13–25)	6519	43.8	21 (12–27)
2022	172	64.9	23 (15–27)	33	47.4	23 (17–27)	15	71.4	21 (17–27)	7651	44.0	20 (12–27)
2023	158	78.1	22 (14–27)	17	37.5	25 (17–27)	24	76.9	22 (15–27)	7123	43.7	21 (12–27)
Total	1020	70.9	21 (13–27)	163	44.3	22 (12–27)	198	70.4	20 (13–27)	55 111	45.5	21 (12–27)

Hospital records were reviewed manually by a trained data entry employee (medical assistant), to determine if the patient met the definitions. In case of doubt, the case was discussed within the study team. For cases in which doubt remained after discussion, the final decision was made by an expert panel including a psychiatrist and an emergency physician.

### Data collection

In this retrospective cohort study, cases were identified for the period 1 January 2016 until 31 December 2023, using hospital management data, electronic health record data through CTcue, and administrative data (see Table 2). The study period was selected based on data availability.

To compare data on our study population with data of emergency department patients of the same age who did not meet the inclusion criteria, additional data from the business intelligence department were used. These data included the number of visits, repeat visits and patient characteristics (age, gender) in all patients aged 12**–**27 years visiting the emergency department in the period 1 January 2016 until 31 December 2023, including patients not fulfilling the inclusion criteria. This allows us to place findings in our study population within a broader context of findings in the general emergency department population of the same age.

### Ethical considerations

The authors assert that all procedures contributing to this work comply with the ethical standards of the relevant national and institutional committees on human experimentation and with the Helsinki Declaration of 1975, as revised in 2013. The study was approved by the Delft University of Technology Human Research Ethics Committee on 27 May 2024 (application number 4249), and by the local Medical Ethics Committee Leiden, Den Haag, Delft (LDD), on 12 February 2024 (reference number N24.009). The study was reviewed and classified as not subject to the Dutch Medical Research Involving Human Subjects Act. The requirement for individual informed consent was formally waived by the LDD Medical Ethics Committee, as the study involved retrospective analysis of pseudonymised, routinely collected clinical data that posed no risk to patients. An opt-out procedure is in place at the hospital: upon registration, all patients are informed that their medical data may be used for scientific research unless they actively object.

### Data processing

All patient visits that did not meet the inclusion criteria were manually removed. Duplicates were deleted. Repeat emergency department visits were marked. For patients without a recorded date of birth in the management file, the date of birth was manually retrieved from hospital records. If the date of birth was not registered in the hospital system, the visit was excluded from this study.

### Data analyses

Descriptive analyses were used to report on the number of suicide attempts, suicidal ideation and non-suicidal self-injury over time, using emergency department visits (not patients) as the analytic unit. Suicide attempts were separated into primary and repeated suicide attempts. Repeat visits for suicide attempt, suicidal ideation or non-suicidal self-injury (as well as for any reason) were identified across the same fixed study period (1 January 2016 to 31 December 2023). Patients were included based on their first recorded visit within this period, with follow-up time varying accordingly: up to 8 years for those presenting in 2016, and less than 1 year for those first presenting in late 2023. Repeat visits were defined as any additional emergency department presentation for the specified reasons (or any reason, for the comparison group) by the same patient within the remaining observation window. Proportions of patients with one or more repeat visits were presented by year of first visit.

To assess trends in suicide attempts, suicidal ideation and non-suicidal self-injury, we estimated incidence rate ratios (IRRs) by using crude (unadjusted) Poisson regression models, with emergency department visits as the unit of analysis. The natural logarithm of the total number of emergency department visits per year was used as an offset term, to account for changes in overall emergency department volume in the same age group. A model with year entered as a continuous variable yielded the overall IRRs reflecting the average annual percent change. Year-on-year IRRs were obtained by modelling year as categorical variables, using 2016 as the reference year.

To identify factors associated with suicide attempt risk (versus other emergency department visits in the same age group), we conducted multivariable logistic regression analysis at the visit level, with emergency department visits as the analytic unit. Predictors included year (continuous), gender, three age categories (12**–**17, 18**–**22 and 23**–**27 years) and a binary indicator for the initial COVID-19 pandemic containment period (16 March to 26 May 2020, when the Dutch Government implemented strict mitigation measures, including school closures, work-from-home mandates and bans on public gatherings). Using backward elimination, we sequentially removed non-significant predictors (*p* > 0.05). The final model included all variables that remained statistically significant after this selection process. No adjustment was applied for clustering by patient: multiple visits by the same individual were treated as independent observations, as the primary interest was in visit-level associations. The analysis was repeated with suicidal ideation and non-suicidal self-injury as dependent variables.

For a subset of 30 patients with a suicide attempt, costs were calculated from a health insurer’s perspective, using patients (not visits) as the analytic unit. This calculation was not feasible for all patients, so we randomly selected ten patients for each of the years 2020, 2021 and 2022. For these patients, the costs of invoiced hospital care products related to the suicide attempt or repeat suicide attempt(s) were calculated by multiplying them by the average tariff in The Netherlands in 2024. Only hospital care was included in the costs; other healthcare and non-healthcare costs were not included. Totals were aggregated per person and per person-year. Resource use unrelated to suicide attempt was manually excluded, such as care provided for other unrelated conditions. Earlier years were excluded because they were not fully captured in the financial database, which was needed to determine resource use. No patients from the year 2023 were included, to have a minimum of 1 year of follow-up for each selected case. Costs are reported in euros in total and per person-year.

All analyses were performed using SPSS version 29.0.0 for Windows (IBM, New York, New York, USA; https://www.ibm.com/support/pages/downloading-ibm-spss-statistics-29).

## Results

### Identified cases by data source

A total of 1381 visits were identified. Through the management data, 1182 visits (85.6%) were included (904 suicide attempts, 113 suicidal ideation, 165 non-suicidal self-injuries). Through the CTcue data, an additional 143 visits (10.4%) were identified for inclusion (93 suicide attempts, 34 suicidal ideation, 16 non-suicidal self-injuries). All included visits were manually checked in the hospital database for repeat visits that had not been picked up through the other sources, resulting in an additional 56 inclusions (4.1%; 23 suicide attempts, 16 suicidal ideation, 17 non-suicidal self-injuries). In total, 1381 records were included in the final analysis (1020 suicide attempts, 163 suicidal ideation, 198 non-suicidal self-injuries).

### Number of youth suicide attempts, suicidal ideation and non-suicidal self-injury

**Fig. 1:**
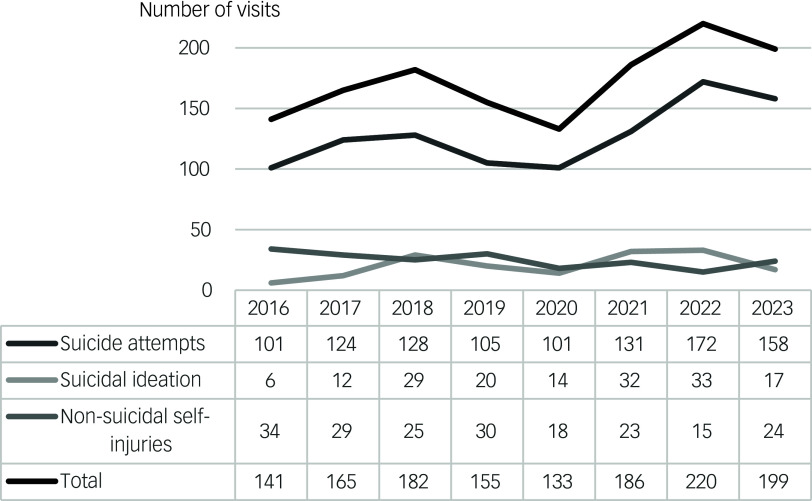
Trend in number of suicide attempts, suicidal ideation and non-suicidal self-injuries. Total number of emergency department visits for 12- to 27-year olds (irrespective of reason) for the years 2016–2023 were, respectively: 6849, 6706, 6628, 7968, 6443, 6705, 7871 and 7322.

The number of suicide attempts increased from 101 in 2016 to 158 in 2023, with the peak in 2022 (*n* = 172) (see Fig. 1). Recorded cases of suicidal ideation without suicide attempt increased from six in 2016 to 17 in 2023, again with the peak in 2022 (*n* = 33). Recorded non-suicidal self-injuries reduced from 34 in 2016 to 24 in 2023.

Poisson regression (Table 3) revealed a significant 4.8% annual increase in suicide attempts per emergency department visit from 2016 to 2023 (IRR = 1.048, 95% CI 1.020–1.077; *p* < 0.001). Year-on-year comparisons showed significantly increased IRRs for suicide attempts for the years 2018, 2021, 2022 and 2023. The highest IRR was observed in 2022 (IRR = 1.482 *v*. 2016, 95% CI 1.159–1.895; *p* = 0.002), with a slight decline in 2023 (IRR = 1.464 *v*. 2016, 95% CI 1.141–1.880; *p* = 0.003).

Poisson regression results for suicidal ideation and non-suicidal self-injuries are provided in Supplementary Table 2. Although these suggest a significant 10.1% annual increase in recorded cases of suicidal ideation and a significant 8.5% annual decrease in recorded cases of non-suicidal self-injury, these findings are likely to be statistically unstable, given the small number of events in these groups (see Table 4).

### Patient characteristics on youth suicide attempts, suicidal ideation and non-suicidal self-injury

The majority of patients with a suicide attempt or non-suicidal self-injury were female. For suicide attempts, 70.9% were female, varying from 55.0 to 78.9% across the years (Table 4).

Logistic regression analysis revealed that female gender was significantly associated with increased odds of suicide attempt (odds ratio 1.603, 95% CI 1.237–2.075; *p* < 0.001); none of the other examined predictors (age category, calendar year or COVID-19 period) demonstrated statistically significant associations in the final model (see Table 5). Logistic regression results for the other outcomes (suicidal ideation and non-suicidal self-injury) are reported in Supplementary Table 3.

**Table 5:** Significant predictors in final logistic regression models for suicide attempts (visit level)

Suicide attempt			
Independent variables	*β* (s.e.)	*p*-value	Odds ratio (95% CI)
Model including all variables, before backward selection			
COVID-19 measures	−0.217 (0.500)	0.665	0.805 (0.302–2.147)
Gender, female^a^	0.480 (0.135)	<0.001	1.616 (1.240–2.104)
Year 2017^b^	0.055 (0.266)	0.838	1.056 (0.627–1.780)
Year 2018^b^	−0.141 (0.255)	0.580	0.869 (0.527–1.431)
Year 2019^b^	−0.270 (0.261)	0.302	0.764 (0.458–1.274)
Year 2020^b^	0.172 (0.302)	0.569	1.188 (0.657–2.146)
Year 2021^b^	−0.178 (0.254)	0.484	0.837 (0.509–1.377)
Year 2022^b^	0.245 (0.256)	0.338	1.278 (0.774–2.111)
Year 2023^b^	0.298 (0.264)	0.259	1.347 (0.803–2.262)
Age group 18–22 years^c^	−0.031 (0.176)	0.859	0.969 (0.687–1.368)
Age group 23–27 years^c^	0.155 (0.184)	0.400	1.167 (0.814–1.673)
Model intercept	0.652 (0.260)	0.012	1.920
Final model, after backward selection			
Gender, female^a^	0.472 (0.132)	<0.001	1.603 (1.237–2.076)
Model intercept	0.721 (0.109)	<0.001	2.056

a. Reference category: male.

b. Reference category: year 2016.

c. Reference category: age group 12–21 years.

### Number of repeated suicide attempts, suicidal ideation and non-suicidal self-injuries

We included 1381 emergency department visits for suicide attempts, suicidal ideation or non-suicidal self-injuries, by 987 patients. Of these, 71.5% (*n* = 706) had one visit and 28.5% (*n* = 281) had two or more visits for the above reasons. Patients with repeat visits made a median of two visits for these reasons per patient (range 2–11). A total of 48.5% of our patients (*n* = 479) had 1 or multiple repeat visits for any reason (not specific to suicide attempt/suicidal ideation/non-suicidal self-injury), corresponding to 873 visits.

In the patient group visiting the emergency department for other reasons than suicide attempt, suicidal ideation or non-suicidal self-injury (*n* = 45 336), 32.9% (18 143) had 1 or more repeat visits during the study period that was not specific to suicide attempt, suicidal ideation or non-suicidal self-injury (see Table 6). This includes all repeat visits, for any reason.

**Table 6:** Trends in repeat visits for youth suicide attempts, suicidal ideation and non-suicidal self-injuries

Year	Number of suicide attempts, suicidal ideation and non-suicidal self-injuries per patient		Repeat visits for suicide attempts, suicidal ideation and non-suicidal self-injuries per patient^a^			Repeat visits by youth (12–27 years), other than suicide attempts, suicidal ideation and non-suicidal self-injuries per patient *N* = 55 111^a^		
	Median (range)	Mean (s.d.)	% (*n*) of repeat visit for suicide attempts, suicidal ideation and non-suicidal self-injuries per patient	% (*n*) visits by female patients^b^	Median age during repeat visits, years (range)	% (*n*) of patients with ≥1 repeat visit	% (*n*) female repeat visitors	Median age of repeat visitors, years (range)
2016	1 (1–4)	1.50 (0.83)	17.0 (24)	81.0 (17)	21 (16–27)	20.4 (1367)	52.5 (718)	22 (12–27)
2017	1 (1–6)	1.82 (1.20)	26.7 (44)	90.9 (40)	23 (14–27)	30.6 (2002)	51.0 (1021)	22 (12–27)
2018	2 (1–7)	2.34 (1.60)	22.5 (41)	92.7 (38)	22 (16–27)	35.3 (2275)	49.8 (1133)	22 (12–27)
2019	2 (1–7)	2.20 (1.56)	32.3 (50)	76.0 (38)	22 (14–27)	36.2 (2828)	48.9 (1382)	22 (12–27)
2020	2 (1–10)	2.98 (2.30)	31.6 (42)	78.6 (33)	22 (14–27)	36.1 (2277)	47.2 (1075)	22 (12–27)
2021	2 (1–11)	2.91 (2.07)	34.9 (65)	90.8 (59)	20 (14–27)	36.8 (2398)	43.4 (1040)	21 (12–27)
2022	1 (1–7)	2.16 (1.70)	28.6 (63)	71.4 (45)	23 (17–27)	31.6 (2420)	41.3 (999)	22 (12–27)
2023	2 (1–10)	3.31 (2.78)	32.7 (65)	89.2 (58)	21 (15–27)	36.2 (2576)	43.9 (1131)	22 (12–27)
Total	2 (1–11)	2.51 (2.01)	28.5 (394)	83.2 (328)	21 (14–27)	32.9 (18 143)	46.8 (8499)	22 (12–27)

a. Proportions of patients with repeat visits are based on variable follow-up time within the study period (2016–2023). Earlier years have longer observation windows. Year-on-year comparisons should be interpreted with caution.

b. Based on 391 visits, because of three missing entries for gender.

### Cost of hospital care

No significant differences in patient and visit characteristics (age, gender, mode of arrival, triage urgency, disposition) were identified for patients included in the cost analysis compared with the other patients with a suicide attempt (see Supplementary Table 4). Median suicide attempt-related costs per patient were €930, ranging from €385 for a single emergency department episode to €33 473 for extensive multi-trauma care, various surgeries and in-hospital admissions (see Table 7). Median cost per person-year was €272, with most patients only having one suicide attempt-related hospital visit in the study period.

**Table 7:** Hospital costs from a health insurer perspective, 2024 price level

Year	Total cost in €		Cost per person-year in €	
	Median (range)	Mean (s.d.)	Median (range)	Mean (s.d.)
2020 (*n* = 10)	2813 (385–14 149)	3457 (4038)	683 (91–3325)	794 (948)
2021 (*n* = 10)	650 (505–33 473)	4512 (10 263)	185 (135–9869)	1348 (3024)
2022 (*n* = 10)	698 (385–20 113)	3909 (6447)	294 (135–7378)	1551 (2423)
Total (*n* = 30)	930 (385–33 473)	3960 (7130)	272 (91–9868)	1231 (2246)

## Discussion

### Discussion on findings

Between 2016 and 2023, 1381 patients visited this emergency department with a suicide attempt, suicidal ideation or non-suicidal self-injury: an average of more than 3 patients per week. The number of suicide attempts increased significantly by 4.8% per year, from 101 in 2016 to 158 in 2023, with a peak in 2022 (IRR = 1.482 *v*. 2016). The majority (71%) of emergency department patients with a suicide attempt were female. Although, in The Netherlands, suicide attempts are more common in female than male youths, the opposite is true for suicides: suicide is more common in male children and adolescents.^4^ Except for gender, no statistically significant factors were identified associated with suicide attempt risk. Although we did not identify a change in suicide attempt presentations during the period with COVID-19 lockdown measures, we cannot determine the potential longer-term impact of the COVID-19 pandemic on the reported trends. Our analysis of the effects of the COVID-19 pandemic was limited to a binary indicator for the short initial lockdown period, and did not constitute a formal interrupted time-series or segmented regression analysis. Consequently, we cannot draw conclusions about longer-term pandemic effects, changes in help-seeking behaviour or the influence of subsequent waves and mitigation measures.

A total of 28.5% of our study population returned to this emergency department for a suicide attempt, suicidal ideation or non-suicidal self-injury during our study period. Our study population visited the hospital more often than other patients in this age category. This aligns well with the international literature. Research from Kapur et al^7^ showed that 13.6% of patients with suicidal behaviour (not specific to youth) returned with suicidal behaviour within 1 year. Our study period included 8 years, with longer follow-up for patients from the earlier years. The proportion of patients with repeat visits in our study (28.5%) should be interpreted with caution, as follow-up time varied: patients from earlier years (e.g. 2016) had longer observation windows than those from later years (e.g. 2023). This likely biased repeat visit proportions downward in later cohorts (see Table 6). Next to the usual referrals, part of our study population (51.3%) had additionally been offered aftercare after visiting the emergency department with suicide attempt, suicidal ideation or non-suicidal self-injury, through a project called ‘Sumona’, which is focused on suicide prevention.^14^

The range in hospital costs was large: €385–€33 473 for suicide attempts. This is not surprising, since the type of injuries also ranged from minor to life-threatening, and with or without repeat events within the study period. However, these costs only represent a minor part of the total societal cost of youth suicide. When taking into account the total years of life lost and earnings forgone, the mean cost of suicide in The Netherlands was previously estimated at $707 645 in 2014 international dollars.^22^

Although the majority of our study cases were identified through the manually collected management data, 14% of cases were missed in this way and were later identified using the CTcue Patient Finder and administrative data from the business intelligence department. If this CTcue search had been used as a primary source, 429 (31.1%) of currently identified cases would have been picked up. However, both require manual screening of patient records to include/exclude cases. Reliably identifying these patients in a non-labour-intensive way remains challenging. Text mining applications^23^ and machine learning^24^ offer promise in suicide research, but have yet to prove their potential for the current purpose.

### Strengths and limitations

We created a reliable database, for which all patient records were manually checked against our selection criteria. It provides insight into the changes in suicide attempts, suicidal ideation and non-suicidal self-injuries over time, including repeat visits and costs. To our knowledge, Dutch data on this level of detail and with this level of reliability is not available from any other source. However, important limitations remain.

The main limitation is that it is challenging to determine which patients fulfil the definition of a suicide attempt, suicidal ideation and non-suicidal self-injury based on data from hospital records, especially when data from a psychiatric evaluation is lacking. Emergency department records from emergency clinicians and other specialists at the emergency department do not always specify patients’ intentions, and can sometimes be interpreted in multiple ways. If reporting practices have changed over time, making it more or less likely to record suicidal intent, this might have influenced the trends we observed. This limitation is inherent in the use of secondary data sources. We may have underestimated the number of suicide-related cases at our emergency department, in cases where suicidal intent was not recorded in the hospital records.

The main limitation of the logistic regression analyses was that the visit-level logistic regression models did not account for clustering within patients with multiple visits, potentially violating the independence assumption and underestimating variance in the estimated odds ratios.

The main limitation of the cost analysis is the limited number of patients included, because of the time-intensive nature of the analysis. Also, included costs are based on insurer tariffs, which may or may not be a good approximation of the true cost of resources. Furthermore, patients can receive care from multiple care providers, including multiple hospitals. Hospital density in The Netherlands is relatively high, so it is not unlikely that patients received care in more than one hospital; for example, for repeat event(s). Therefore, it is likely that we underestimated the hospital costs involved in the care for suicide attempt patients by including care provided in only one hospital.

Furthermore, the population living in the catchment area of the study hospital differs from those in other regions in The Netherlands and abroad. For instance, the ethnic composition and the hospital’s central urban location may influence patterns of youth suicide attempts, suicidal ideation and non-suicidal self-injuries. The HMC emergency department sees a relatively high number of psychiatric presentations compared with other hospitals in the region, making it a particularly relevant setting for examining trends in suicidal behaviour in an urban emergency care context. However, these characteristics limit the representativeness of the findings, and the results cannot be generalised to other non-urban or demographically different regions.

Finally, we did not study the causes of the trends we observed; this would require further research. Context on the societal and policy changes, with an impact on child and adolescent mental health services, in 2016–2023 will be published in a companion paper.

### Implications

A substantial number of young people visit the emergency department for suicide-related events, affecting both them and their families, as well as emergency department capacity and costs. More understanding is needed on successful strategies for the prevention and treatment of youths with suicidal thoughts and behaviours. Additionally, emergency department staff, hospital paediatricians and consultants in internal medicine should be knowledgeable on how to communicate and provide appropriate care to this vulnerable patient population.^25^

Emergency departments could play an important role in secondary prevention of suicide attempts,^13^ for example, by ensuring their patients receive assessment and a care plan from healthcare professionals before discharge from the department, and establishing strong care coordination agreements with providers specialised in suicidal behaviour. A good local example is the Sumona project, which provides case management without care waiting lists for people with suicidal behaviour, and has achieved promising results in this respect.^26^ However, challenges remain. The cost-effectiveness of programmes like Sumona is largely unknown, and financial sustainability has not been adequately secured to guarantee their continuity and impact over time.^27^ Furthermore, although such programmes can assist patients in finding the right type of care, limited availability and long waiting lists for child and adolescent mental health services may reduce the effectiveness of the overall care trajectory.

More generally, a lack of high-quality economic evidence on the cost-effectiveness of suicide prevention measures and treatment strategies limits opportunities for evidence-based decision-making in this space. We hope the current paper provides a step in the right direction. Since hospital-invoiced costs represent only a small part of healthcare costs for these patients, future analyses should broaden this scope to include societal costs within and outside the healthcare sector.

Finally, consistent, national registration of youth suicide attempts, suicidal ideation and non-suicidal self-injuries could help provide insight into trends, the size of the problem and its cost. This could inform and potentially justify future investments in care, support and education with respect to suicide prevention, to reduce the number of suicide and self-injury-related events.

## Supporting information

10.1192/bjo.2026.11031.sm001 van der Linden et al. supplementary material van der Linden et al. supplementary material

## Data Availability

An aggregate version of the data that support the findings of this study is available via the 4TU.ResearchData repository: https://doi.org/10.4121/d3cab4d1-bc20-4d04-ace2-f763a4ba6ffe.

## Supplementary material

The supplementary material is available online at https://doi.org/10.1192/bjo.2026.11031
