# Supplementary material for: Youth visiting the emergency department after a suicide attempt, suicidal ideation or non-suicidal self-injury: trends, repeat visits and costs
Source: BJPsych Open. 2026 Apr 28;12(3):e117. doi: 10.1192/bjo.2026.11031 (PMC13122344; doi:10.1192/bjo.2026.11031)
Supplement: van der Linden et al. supplementary material [file S205647242611031Xsup001.docx]

## Supplementary Material

Table S1 Diagnosis codes used to identify patients in the electronic medical records through CTCue

| **Diagnosis code** | **Original description (in Dutch)** | **English translation** |
| --- | --- | --- |
| F10-F19 | Psychische stoornissen en gedragsstoornissen door gebruik van psychoactieve middelen | Mental and behavioral disorders due to psychoactive substance use |
| F20-F29 | Schizofrenie, schizotypische stoornissen en waanstoornissen | Schizophrenia, schizotypal and delusional disorders |
| F31.1 | Bipolaire affectieve stoornis, huidige episode manisch zonder psychotische symptomen | Bipolar affective disorder, current episode manic without psychotic symptoms |
| F31.3 | Bipolaire affectieve stoornis, huidige episode licht of matig depressief | Bipolar affective disorder, current episode mild or moderate depression |
| F31.4 | Bipolaire affectieve stoornis, huidige episode ernstig depressief zonder psychotische symptomen | Bipolar affective disorder, current episode severe depression without psychotic symptoms |
| F32 | Depressieve episode | Depressive episode |
| F41.0 | Paniekstoornis [episodische paroxismale angst] | Panic disorder [episodic paroxysmal anxiety] |
| F41.2 | Gemengde angststoornis en depressieve stoornis | Mixed anxiety and depressive disorder |
| F41.9 | Angststoornis niet gespecificeerd | Anxiety disorder, unspecified |
| F43 | Reactie op ernstige stress en aanpassingsstoornissen | Reaction to severe stress, and adjustment disorders |
| F50.0 | Anorexia nervosa | Anorexia nervosa |
| F60.9 | Persoonlijkheidsstoornis niet gespecificeerd | Personality disorder, unspecified |
| F91.9 | Gedragsstoornis niet gespecificeerd | Behavioral disorder, unspecified |
| F99 | Psychische stoornis, niet nader omschreven | Mental disorder, not otherwise specified |
| S51.9 | Open wond van onderarm, deel niet gespecificeerd | Open wound of forearm, part unspecified |
| T36-T50 | Vergiftiging door geneesmiddelen en biologische stoffen | Poisoning by drugs, medicaments and biological substances |
| T53.4 | Toxisch gevolg van dichloormethaan | Toxic effect of dichloromethane |
| T71 | Asfyxie | Asphyxia |
| R45.2 | Zich ongelukkig voelen | Feeling unhappy |
| R45.7 | Toestand van emotionele shock en stress, niet gespecificeerd | State of emotional shock and stress, unspecified |
| R45.8 | Overige symptomen betreffende emotionele toestand | Other symptoms concerning emotional state |
| X60-X84 | Opzettelijk zichzelf schade toebrengen | Intentional self-harm |
| Z53.2 | Verrichting niet uitgevoerd wegens beslissing van patiënt om overige en niet gespecificeerde redenen | Procedure not carried out due to patient's decision for other and unspecified reasons |
| Z60.9 | Probleem verband houdend met sociale omgeving, niet gespecificeerd | Problem related to social environment, unspecified |
| 0000085160 | Tentamen suicidii door middel van autointoxicatie | Suicide attempt by means of self-poisoning |
| 0000092139 | Letsel door tentamen suicide | Injury from suicide attempt |
| CS00000752 | Risico op suicidaal gedrag | Risk of suicidal behavior |
| CS00000575 | Risico op suicide | Risk of suicide |
| 0000035930 | Autointoxicatie | Self-poisoning |
| XXI | Uitwendige oorzaken van ziekte en sterfte: opzettelijke zelfbeschadiging | External causes of morbidity and mortality: intentional self-harm |

Table S2 Poisson regression results, suicidal ideation and non-suicidal self-injury, using the natural logarithm of the total number of ED visits in the same age group as an offset*

| **Parameter** | **B** | **Std. Error** | **95% Wald Confidence Interval**  **(lower ; upper)** | **Hypothesis Test Wald Chi-Square** | **df** | **Significance** | **Exp(B)** | **95% Wald Confidence Interval for Exp(B) (lower ; upper)** |
| --- | --- | --- | --- | --- | --- | --- | --- | --- |
| **Suicidal ideation** | | | | | | | | |
| **Evaluation of yearly trend, using a continuous year variable** | | | | | | | | |
| Intercept | -199.300 | 70.188 | -336.866 ; -61.735 | 8.063 | 1 | 0.005 | <0.001 | 0.000 ; 0.000 |
| Year | 0.096 | 0.035 | 0.028 ; 0.164 | 7.599 | 1 | 0.006 | 1.101 | 1.028 ; 1.178 |
| **Evaluation of year-on-year incidence rate rations, comparing individual years against a reference (2016)** | | | | | | | | |
| Intercept | -6.065 | 0.2425 | -6.540 ; -5.589 | 625.288 | 1 | <0.001 | 0.002 | 0.001 ; 0.004 |
| 2016 | -0.975 | 0.4749 | -1.906 ; -0.045 | 4.220 | 1 | 0.040 | 0.377 | 0.149 ; 0.956 |
| 2017 | -0.261 | 0.3770 | -1.000 ; 0.478 | 0.480 | 1 | 0.488 | 0.770 | 0.368 ; 1.612 |
| 2018 | 0.633 | 0.3055 | 0.034 ; 1.232 | 4.295 | 1 | 0.038 | 1.883 | 1.035 ; 3.427 |
| 2019 | 0.078 | 0.3299 | -0.569 ; 0.724 | 0.055 | 1 | 0.814 | 1.081 | 0.566 ; 2.063 |
| 2020 | -0.067 | 0.3609 | -0.775 ; 0.640 | 0.035 | 1 | 0.852 | 0.935 | 0.461 ; 1.897 |
| 2021 | 0.720 | 0.3001 | 0.131 ; 1.308 | 5.748 | 1 | 0.017 | 2.053 | 1.140 ; 3.698 |
| 2022 | 0.590 | 0.2985 | 0.005 ; 1.175 | 3.910 | 1 | 0.048 | 1.805 | 1.005 ; 3.240 |
| 2023 | 0 | . | . | . | . |  | 1 | . |
|  | | | | | | | | |
| **Non-suicidal self-injury** | | | | | | | | |
| **Evaluation of yearly trend, using a continuous year variable** | | | | | | | | |
| Intercept | 174.463 | 63.071 | 50.847 ; 298.079 | 7.652 | 1 | 0.006 | 5.866*e^75^ | 1.210*e^22^ ; 2.845*e^129^ |
| Year | -0.089 | 0.031 | -0.150 ; -0.028 | 8.154 | 1 | 0.004 | 0.915 | 0.860 ; 0.972 |
| **Evaluation of year-on-year incidence rate rations, comparing individual years against a reference (2016)** | | | | | | | | |
| Intercept | -5.720 | 0.2041 | -6.120 ; -5.320 | 785.227 | 1 | <0.001 | 0.003 | 0.002 ; 0.005 |
| 2016 | 0.414 | 0.2666 | -0.108 ; 0.937 | 2.415 | 1 | 0.120 | 1.513 | 0.897 ; 2.552 |
| 2017 | 0.276 | 0.2760 | -0.265 ; 0.817 | 1.002 | 1 | 0.317 | 1.318 | 0.768 ; 2.264 |
| 2018 | 0.140 | 0.2858 | -0.420 ; 0.700 | 0.239 | 1 | 0.625 | 1.150 | 0.657 ; 2.014 |
| 2019 | 0.138 | 0.2739 | -0.399 ; 0.675 | 0.254 | 1 | 0.614 | 1.148 | 0.671 ; 1.964 |
| 2020 | -0.161 | 0.3118 | -0.772 ; 0.450 | 0.266 | 1 | 0.606 | 0.852 | 0.462 ; 1.569 |
| 2021 | 0.044 | 0.2918 | -0.527 ; 0.616 | 0.023 | 1 | 0.879 | 1.045 | 0.590 ; 1.852 |
| 2022 | -0.543 | 0.3291 | -1.188 ; 0.102 | 2.722 | 1 | 0.099 | 0.581 | 0.305 ; 1.107 |
| 2023 | 0 | . | . | . | . | . | 1 | . |

* While these suggest a significant 10.1% annual increase in recorded cases of suicidal ideation and a significant 8.5% annual decrease in recorded cases of non-suicidal self-injury, these findings are likely to be statistically unstable given the small number of events in these groups (see Table 4 in the main manuscript).

Table S3 Significant predictors in final logistic regression models for suicidal ideation and non-suicidal self-injury (visit level)

| **Suicidal ideation** | | | |
| --- | --- | --- | --- |
| **Independent variables** | **Beta (S.E.)** | **P** | **OR (95% CI)** |
| ***Model including all variables, before backward selection*** | | | |
| Covid measures | 0.773 (0.593) | 0.192 | 2.167 (0.678 ; 6.927) |
| Gender, Female^1^ | 0.195 (0.158) | 0.218 | 1.215 (0.891 ; 1.655) |
| Year 2017^2^ | 0.886 (0.533) | 0.096 | 2.425 (0.854 ; 6.888) |
| Year 2018^2^ | 1.771 (0.485) | <0.001 | 5.876 (2.273 ; 15.191) |
| Year 2019^2^ | 1.230 (0.500) | 0.014 | 3.420 (1.283 ; 9.118) |
| Year 2020^2^ | 0.948 (0.548) | 0.084 | 2.580 (0.881 ; 7.555) |
| Year 2021^2^ | 1.918 (0.481) | <0.001 | 6.805 (2.649 ; 17.478) |
| Year 2022^2^ | 1.812 (0.480) | <0.001 | 6.124 (2.388 ; 15.701) |
| Year 2023^2^ | 1.206 (0.509) | 0.018 | 3.341 (1.232 ; 9.063) |
| Age group 18 – 22 years^3^ | 0.898 (0.246) | <0.001 | 2.456 (1.515 ; 3.981) |
| Age group 23 – 27 years^3^ | 0.802 (0.245) | 0.001 | 2.229 (1.380 ; 3.602) |
| Model intercept | -8.017 (0.497) | <0.001 | 0.000 |
| ***Final model, after backward selection*** | | | |
| Year 2018^2^ | 0.776 (0.222) | <0.001 | 2.173 (1.407 ; 3.357) |
| Year 2021^2^ | 0.917 (0.214) | <0.001 | 2.501 (1.643 ; 3.808) |
| Year 2022^2^ | 0.809 (0.212) | <0.001 | 2.245 (1.481 ; 3.404) |
| Age group 18 – 22 years^3^ | 0.924 (0.246) | <0.001 | 2.519 (1.557 ; 4.076) |
| Age group 23 – 27 years^3^ | 0.813 (0.244) | <0.001 | 2.265 (1.397 ; 3.639) |
| Model intercept | -6.938 (0.234) | <0.001 | 0.001 |
| **Non-suicidal self-injury** | | | |
| **Independent variables** | **Beta (S.E.)** | **P** | **OR (95% CI)** |
| ***Model including all variables, before backward selection*** | | | |
| Covid measures | 0.106 (0.634) | 0.867 | 1.112 (0.321 ; 3.851) |
| Gender, Female^1^ | 1.265 (0.168) | <0.001 | 3.541 (2.547 ; 4.924) |
| Year 2017^2^ | -0.052 (0.259) | 0.840 | 0.949 (0.571 ; 1.577) |
| Year 2018^2^ | -0.190 (0.270) | 0.481 | 0.827 (0.487 ; 1.403) |
| Year 2019^2^ | -0.183 (0.257) | 0.476 | 0.833 (0.503 ; 1.378) |
| Year 2020^2^ | -0.477 (0.315) | 0.130 | 0.620 (0.334 ; 1.151) |
| Year 2021^2^ | -0.237 (0.276) | 0.390 | 0.789 (0.459 ; 1.355) |
| Year 2022^2^ | -0.802 (0.315) | 0.011 | 0.448 (0.242 ; 0.832) |
| Year 2023^2^ | -0.306 (0.276) | 0.268 | 0.737 (0.429 ; 1.265) |
| Age group 18 – 22 years^3^ | 0.439 (0.187) | 0.019 | 1.551 (1.076 ; 2.238) |
| Age group 23 – 27 years^3^ | -0.209 (0.204) | 0.305 | 0.811 (0.544 ; 1.210) |
| Model intercept | -6.303 (0.258) | <0.001 | 0.002 |
| ***Final model, after backward selection*** | | | |
| Gender, Female^1^ | 1.262 (0.168) | <0.001 | 3.531 (2.541 ; 4.906) |
| Year 2022^2^ | -0.601 (0.269) | 0.026 | 0.548 (0.324 ; 0.930) |
| Age group 18 – 22 years^3^ | 0.569 (0.144) | <0.001 | 1.767 (1.332 ; 2.343) |
| Model intercept | -6.620 (0.162) | <0.001 | 0.001 |

Abbreviations: S.E., Standard Error; OR, Odds Ratio; CI, Confidence Interval.

^1^ Reference category: Male; ^2^ Reference category: Year 2016; ^3^ Reference category: Age group 12 – 21 years.

Table S4 Descriptives of the subset of patients included in the cost analysis versus the other patients with a suicide attempt

|  | **Cost analyses selected cases n=30** | **Cohort not selected for cost analyses**  **N=1020 – 30 = 990** | **P-value** | **Odds Ratio** | **OR (95% CI)** |
| --- | --- | --- | --- | --- | --- |
| Age mean, years | 20.8 | 21.4 | 0.398 |  | -0.778; 1.956 |
| Gender n (%)  Male  Female | 6 (20.0)  24 (80.0) | 253 (25.6)  737 (74.4) | 0.491 | 1.373 | 0.555; 3.397 |
| Arrival by ambulance n (%) | 22 (73.3) | 632 (63.8) | 0.285 | 1.558 | 0.686; 3.535 |
| Triage urgency^1^ n (%)  Non-urgent  Urgent | 1 (3.7)  26 (96.3) | 23 (2.6)  855 (97.4) | 0.730 | 0.699 | 0.091; 5.278 |
| Disposition^1^ n (%)  Discharged home  Admission | 8 (29.6)  19 (70.4) | 266 (30.3)  612 (69.7) | 0.941 | 1.032 | 0.446; 2.388 |

^1^Triage urgency and disposition based on 905 cases, due to 115 (11.3%) missing values.
